# Incidental Finding of Synovial Osteochondromatosis: A Case Report

**DOI:** 10.7759/cureus.28051

**Published:** 2022-08-16

**Authors:** Siya S Bhagat, Jessi Hammond, Danielle M Fuller-Sincock, Syeda B Zehra

**Affiliations:** 1 Physical Medicine and Rehabilitation, Upstate University Hospital, Syracuse, USA; 2 Orthopedics, Dow University of Health Sciences, Civil Hospital, Karachi, PAK

**Keywords:** chondrocyte, synovium, degenerative knee, loose bodies, osteoarthritis, synovial osteochondromatosis

## Abstract

Synovial osteochondromatosis (SOC) is a rare, benign condition of unknown etiology characterized by the formation of cartilaginous loose bodies. This often leads to early osteoarthritis with decreased range of motion and pain. Clinical presentation most often is reported as monoarticular pain affecting the knee or less commonly the hip shoulder or elbow. Diagnosis can be confirmed on x-ray imaging where the characteristic synovial bodies will be identified. Management with early debridement of the synovium will often be curative but more complex cases may require further surgical management.

We present a 33-year-old male presented with localized left knee pain. Given the history of new-onset pain, left knee x-rays were obtained that revealed a displaced patella with multiple ossific densities around the left knee which were suspicious for a new diagnosis of SOC.

Due to its nonspecific symptoms and imaging, the diagnosis of SOC is often delayed or missed. Therefore, prompt diagnosis and treatment are important in order to avoid irreversible cartilage destruction in the joint, prevent the development of chronic pain, and reduce the risk of malignant transformation.

## Introduction

Synovial osteochondromatosis (SOC) clinically presents as a constellation of symptoms that closely mirror traditional joint arthropathies such as osteoarthritis in their temporal course but differ in their histopathology and clinical progression. It is readily contrasted with other joint arthropathies by the distinctive bony lesions found on the synovial membrane of the affected joint. SOC typically initially presents in the fifth decade of life and typically requires plain radiographs for diagnosis [1]. Initial symptoms include chronic knee pain, decreased range of motion, crepitus on range of motion, and joint line tenderness [1,2]. Occasionally further imaging with MRI or CT scans may be required to identify the characteristic bony lesions that seed the synovium of the joint.

SOC is a benign disorder of the synovial lining typically affecting the knee joint but which also may affect the hip elbow or shoulders. The pattern of the presentation can be classified as primary; occurring without evidence of joint pathology, or secondary; which typically accompanies the development of degenerative joint disease [3]. A third rarer subtype has been described affecting bursae and tendon sheaths [3]. No strict consensus exists with regards to the definitive etiology of any of these subtypes however the pathophysiology of SOC in its later stages is fairly well understood. Early detection may allow for management with arthroscopic debridement, but if severe joint degeneration occurs arthroplasty may not be avoidable [4]. Once treated the rate of recurrence is quite low.

## Case presentation

A 33-year-old male presented to the emergency department with seizures. After medical stabilization, the patient was transferred to inpatient rehabilitation to manage residual functional deficits. During his stay, the patient reported localized left knee pain. This pain was unfamiliar compared to the arthritic symptoms he had for years. On physical exam, the patient demonstrated non-specific findings such as painful range of motion, bilateral deformity of the knees, and positive patellar tilt and patellofemoral apprehension tests.

**Figure 1:**
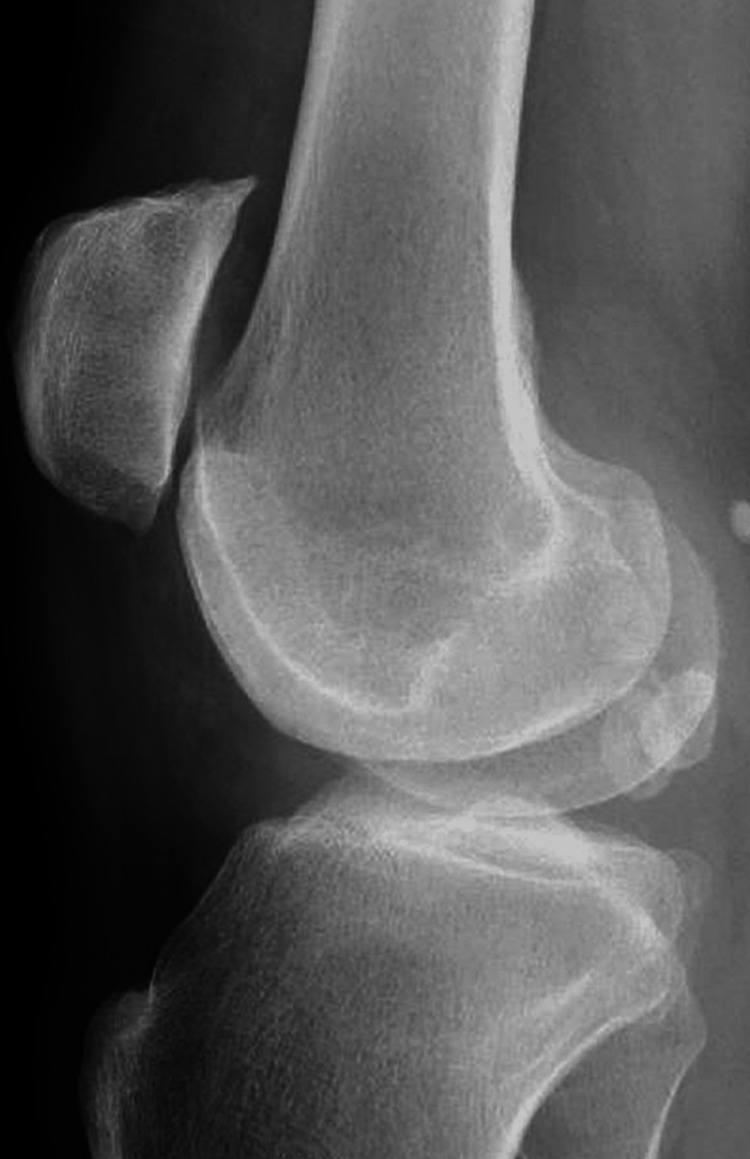
Lateral view of the left knee

**Figure 2:**
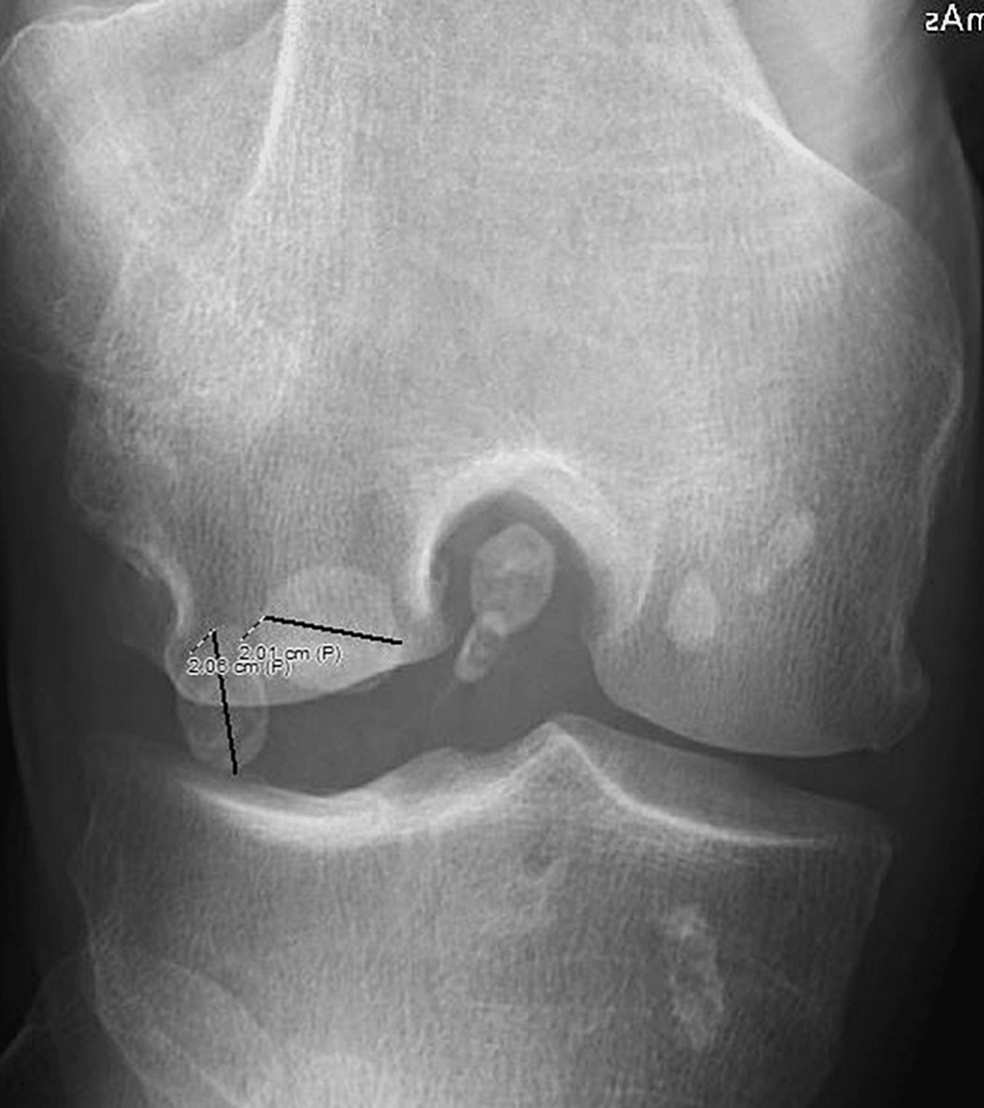
Flex view of the left knee

**Figure 3:**
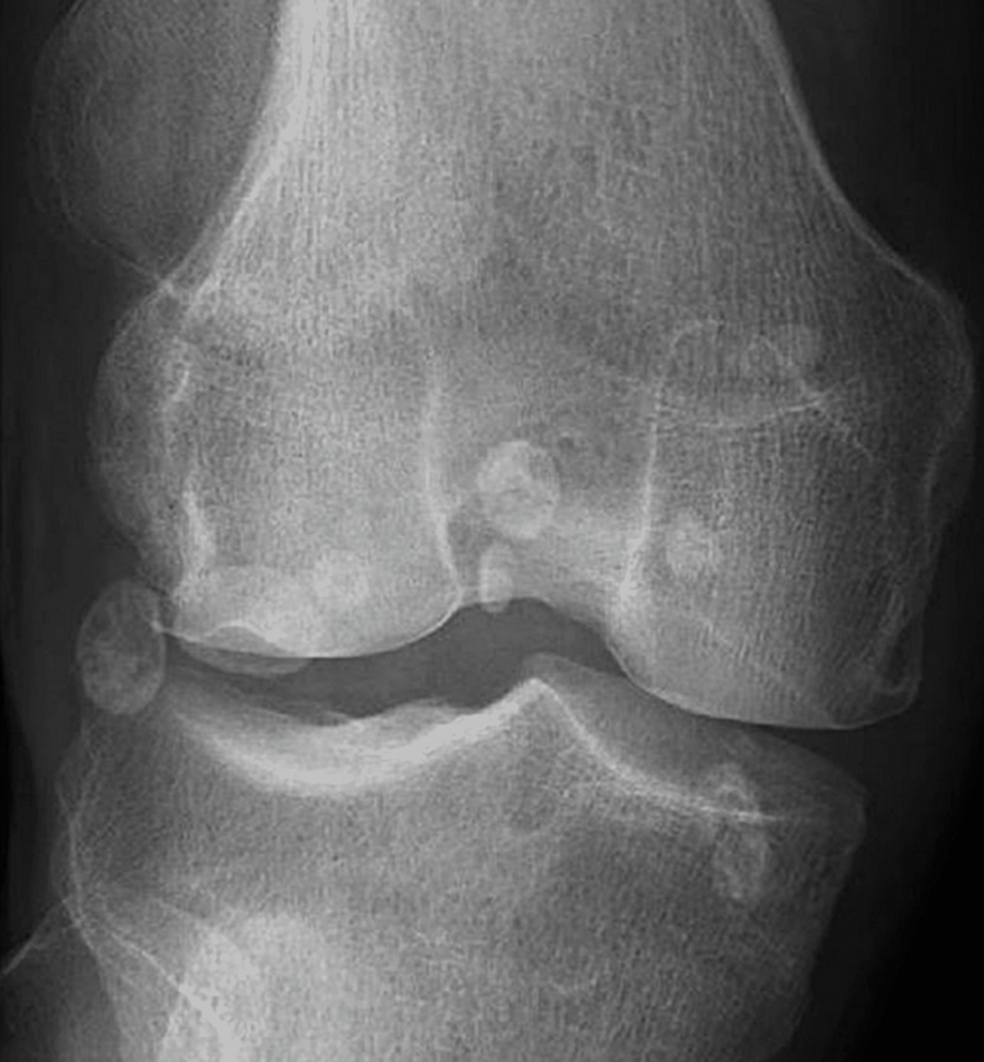
Anteroposterior view of the left knee

Given this history and new-onset pain, x-rays of the left knee were obtained that were expected to reveal worsening arthritis. Instead, they revealed a displaced patella with multiple ossific densities around the left knee, which were suspicious for a new diagnosis of SOC as can be seen in Figures 1-3. The patient received a corticosteroid injection and was instructed to follow up in the outpatient clinic. His steroid injection only provided limited relief of symptoms over the next several weeks. As his pain gradually returned the patient sought further management, he was seen as an outpatient and after failing conservative management was taken to the operating room (OR) for arthroscopy and partial lateral meniscectomy along with chondroplasty left retinacular release and synovectomy with debridement of the patellofemoral medial and lateral compartments. Management with arthroscopy ultimately failed, and the patient required left knee arthroplasty for definitive management.

## Discussion

SOC can be classified as primary or secondary and can be differentiated based on pathological and radiological findings [1,4]. Primary SOC is an idiopathic neoplastic process that occurs with no underlying joint pathology while secondary SOC is associated with underlying arthritis, trauma, infection, or degenerative changes [1,3]. The etiology of SOC is hypothesized to be a form of metaplasia with synovial cells turning into chondrocytes. The inciting stimulus is thought to be due to the overactivity of native pluripotent stem cells that are present in the “transitional zone” between the synovial membrane and articular cartilage [1,5,6]. Milgram described three phases in the disease course of SOC [5]. The first stage consists of an active inflammatory state with metaplasia but no development of loose bodies [1-3,6-8]. Patients are often asymptomatic or present with pain and swelling of the affected joint. The second stage involves the proliferation of chondrocytes with the formation of loose bodies that range from a few millimeters to centimeters in size [1-3,6-8]. The loose bodies cause mechanical symptoms leading to a restricted range of motion and symptoms like joint locking, crepitus, stiffness, as well as recurrent joint effusions [1]. The final stage involves a decrease in synovial inflammation with the ossification of loose bodies [1-3,6-8].

To diagnose SOC, plain radiographs remain an initial imaging modality showing joint effusions and ossified loose bodies. However, if ossification has not yet occurred, the loose bodies can be missed with an x-ray. Therefore, if an x-ray shows non-specific findings, CT or MRI are often required [4]. No specific laboratory abnormalities are found in primary SOC; however, some derangements can be indicative of secondary SOC due to the presence of an underlying pathologic process [9].

The differential diagnosis of SOC is vast and includes conditions like osteoarthritis, rheumatoid arthritis, gout, pseudogout, torn ligament, and avulsion fracture. One important differential is also malignancy, specifically chondrosarcoma or synovial cell sarcoma, which is especially important to consider since SOC has the potential to undergo malignant conversion [4,9]. Many of these potential diagnoses can be excluded when patient history, serum laboratory studies, and imaging are taken into account.

The most supported treatment for SOC is arthroscopic excision of the loose bodies with or without removal of the associated synovial membrane [2,3,10]. Removal of the synovial membrane does not appear to be beneficial. However, could be needed if the disease recurs [10]. Radiation and chemotherapy are usually not needed as surgical removal tends to be sufficient to treat and prevent a recurrence [10]. Without surgical excision, the long-standing disease eventually leads to joint degeneration. In cases with severe knee joint degeneration, total knee replacement has been found to treat SOC [3,11]. Additionally, there is a small risk of malignant transformation to chondrosarcoma [2]. As of 2010, only 33 cases have been identified, all of which involved recurrent disease [2]. Since SOC has the potential to spread to extra-articular structures, it would further support removing the loose bodies upon diagnosis as this could further limit damage to the joint [3].

## Conclusions

SOC requires a high index of suspicion in order to be effectively managed, unfortunately, its close resemblance to osteoarthritis can often lead a provider to underestimate the rapidly progressive course of this disease. Treatment with debridement and synovectomy can often be curative but only as long as the underlying joint maintains its own structural integrity. We, therefore, recommend close follow-up and carefully considering imaging options available when managing progressive arthritis especially when conservative management fails to arrest disease progression. In extreme cases, as presented in this paper management may ultimately require total joint replacement.
